# Phenotype-Aligned Metabolomics Identifies Plasma Arginine as a Candidate Predictor of Delayed Cerebral Ischemia after Aneurysmal Subarachnoid Hemorrhage

**DOI:** 10.1007/s12975-026-01421-0

**Published:** 2026-03-25

**Authors:** Krzysztof Urbanowicz, Karol Wiśniewski, Mikołaj Opiełka, Michał Bieńkowski, Marta Popęda, Oliwier Krajewski, Ernest J. Bobeff, Karol Zaczkowski, Bartosz Szmyd, Dariusz J. Jaskólski, Ryszard T. Smoleński

**Affiliations:** 1 Department of Biochemistry, Medical University of Gdańsk, Gdańsk, Poland; 2 Department of Neurosurgery and Neurooncology, Medical University of Łódź, Barlicki University Hospital, Łódź, Poland; 3 Department of Pathomorphology, Medical University of Gdańsk, Gdańsk, Poland

**Keywords:** Aneurysmal subarachnoid hemorrhage, Delayed cerebral ischemia, Metabolomics, Phenotype alignment, Predictor discovery, Predictive biomarkers

## Abstract

Delayed cerebral ischemia (DCI) affects approximately 30% of aneurysmal subarachnoid hemorrhage (aSAH) survivors and is a leading cause of morbidity and mortality post-aSAH. Current risk assessment relies on radiographic severity scores that stratify populations based on hemorrhage characteristics but cannot predict DCI onset for individual patients. No plasma-based biomarkers for DCI prediction currently exist. We used liquid chromatography – mass spectrometry to perform plasma metabolomics in 60 aSAH patients (50% female, 30 DCI, 30 No-DCI) with longitudinal sampling until 7 days post-aSAH. Samples were retrospectively aligned to individual DCI onset times per patient, isolating 24–48 h and 48–72 h pre-event windows. Arginine emerged as the sole FDR-significant metabolite 24–48 h before DCI (q = 0.022, 39% depletion) and ranked #1 of 2,022 metabolites across three statistical methods (Student’s t-test, Welch’s t-test, Mann-Whitney U test, *p* = 10⁻⁵ to 10⁻⁴). Zero metabolites achieved FDR significance at 48–72 h, confirming temporal specificity. Independent logistic regression validated predictive performance (odds ratio 0.22, *p* = 0.001, AUC 0.808, 95% CI: 0.647–0.945), outperforming modified Fisher radiographic score (AUC 0.718, 95% CI: 0.593–0.830). The combination model of arginine and modified Fisher score performed best with AUC 0.856 (95% CI: 0.733–0.955), with arginine as the dominant factor. Mechanistic investigation revealed FDR-significant depletion of arginine/ornithine (q = 0.011) and glutamine/glutamate ratios (q = 0.011), consistent with increased arginase activity competing with nitric oxide synthesis. Plasma arginine represents a candidate single-molecule biomarker for DCI with a clinically actionable pre-event detection window, pending prospective validation. Temporal alignment to individual DCI onsets enabled detection of pre-event metabolic changes 24–48 h before symptom onset.

**Supplementary Information:**

The online version contains supplementary material available at 10.1007/s12975-026-01421-0.

## Introduction

Delayed cerebral ischemia (DCI) develops in 20–30% of aneurysmal subarachnoid hemorrhage (aSAH) survivors between days 4–14 post-hemorrhage and represents a leading cause of morbidity and mortality in this population [1–3]. Among patients who develop DCI, approximately 30–40% experience death or permanent neurological deficits [4]. Current risk assessment relies primarily on radiographic severity scores, including Fisher, modified Fisher (mFisher), and VASOGRADE scales that stratify patients based on initial hemorrhage characteristics and clinical severity [4–6].

While effective for identifying high-risk populations, these approaches cannot predict individual DCI onset timing. Furthermore, laboratory-based biomarkers for individual-level DCI prediction remain absent despite substantial research efforts. This gap is clinically significant: patients who will develop DCI are clinically indistinguishable from those who will not during the hours to days preceding symptom onset, with identical neurological examinations, vital signs, and monitoring parameters providing no warning. A pre-symptomatic detection window would enable targeted interventions before irreversible ischemic injury. To address this need, extensive research has investigated blood-based biomarkers for DCI and vasospasm prediction, with systematic reviews cataloging over 700 candidate molecules spanning inflammatory markers, hemostatic factors, matricellular proteins, and genetic polymorphisms. Despite this substantial body of work, no validated blood-based biomarker has achieved clinical implementation for individual-level DCI prediction [7, 8]. Multiple studies have examined metabolomics in aSAH and DCI, predominantly employing cerebrospinal fluid (CSF) analysis in patients with external ventricular drains or lumbar punctures [9–14]. These investigations have identified arginine pathway dysregulation as a consistent feature of DCI pathophysiology. Arginase-1, released into CSF from lysed erythrocytes following hemorrhage, depletes arginine pools through increased enzymatic activity [15, 16]. Zimmermann et al. demonstrated that CSF arginine/ornithine ratios below 2.71 at disease onset predicted cerebral vasospasm syndrome with 86.7% sensitivity and 72.2% specificity, correlating with poor functional outcomes [15]. Weller et al. confirmed that CSF arginine/ornithine ratio independently predicted clinical outcome at 3 months when added to established prognostic scores [16]. Additional CSF studies have implicated dimethylarginines (ADMA, SDMA) as endogenous nitric oxide synthase (NOS) inhibitors associated with vasospasm severity [17, 18].

However, CSF sampling is inherently limited: it requires invasive procedures (external ventricular drain or lumbar puncture), restricts serial monitoring, and is not feasible in all patients. Moreover, most prior studies employed fixed-timepoint sampling designs, collecting samples at predetermined calendar days (e.g., Days 3, 5, 7 post-aSAH) regardless of individual DCI timing. This approach creates temporal heterogeneity: a “Day 5” sample might represent 12 h pre-DCI for one patient, 72 h for another, or imminent DCI for a third. Such heterogeneity may obscure pre-event metabolic signals.

Plasma-based biomarkers would offer substantial advantages: minimally invasive sampling, compatibility with serial monitoring, and integration into standard workflows. Recent plasma and serum metabolomics investigations have examined DCI-associated metabolic changes. Chikh et al. employed combined plasma and CSF metabolomics at admission (first 24 h post-aSAH), identifying a 20-metabolite predictive panel with arginine pathway dysregulation detected through enrichment analysis [19]. Snider et al. demonstrated that serum glutamate levels measured at fixed timepoints (days 0–3, 5–7, 14) correlated with cerebral ischemia and poor neurological outcomes [20]. Yet individual plasma metabolites suitable for clinical assay development have not been identified in these or other comprehensive plasma or serum metabolomics investigations employing fixed-timepoint designs [19–21].

A biological premise suggests a solution: metabolic changes likely precede clinical manifestation, creating a detectable pre-DCI window where intervention remains feasible. We hypothesized that retrospective alignment of longitudinal plasma samples to individual DCI onset times would reduce metabolic heterogeneity and enable detection of subtle pre-event metabolic changes. Retrospective temporal alignment stratifies samples according to their temporal relationship to DCI onset (e.g. 1 day and 2 days pre-event), with No-DCI controls matched on post-aSAH time. This stratification creates comparison groups focused on isolating metabolic signatures preceding DCI onset.

The primary aim of this study was to identify plasma metabolic predictors of DCI in the days before its onset through comprehensive metabolomics screening. This approach is generalizable to any condition with discrete clinical events, requires no additional experimental cost beyond routine longitudinal sampling, and is feasible in acute hospital settings such as neurocritical care units where such sampling is standard. Our discovery-phase study addresses the unmet clinical need for plasma-based DCI prediction while demonstrating how temporal alignment of longitudinal samples can isolate clinically actionable metabolic signals in the pre-event window.

## Materials and Methods

### Participants

This single-center prospective observational study enrolled 101 aSAH patients (41 DCI) at Norbert Barlicki University Clinical Hospital in Łódź based on inclusion and exclusion criteria available in Supplementary Data S1. All aSAH patients were diagnosed and treated according to a standardized regimen reported previously [22]. On admission, the patients were assessed using the Hunt and Hess scale and the Glasgow Coma Scale. aSAH was diagnosed using computed tomography (CT), followed by CT angiography and digital subtraction angiography (DSA) for aneurysm evaluation. Radiological grading was performed using the Modified Fisher Scale. Therapeutic decision (microsurgical or endovascular procedure) was made by an interdisciplinary team.

Delayed cerebral ischemia was determined according to consensus criteria as a new neurological state/consciousness deterioration, such as a decrease of ≥ 2 on the Glasgow Coma Scale or appearance of new focal neurological deficits, lasting for at least one hour [23]. DCI diagnosis was confirmed after exclusion of all other possible causes, including procedure-related complications, rerupture, or increase in intracranial pressure. In case of DCI, transcranial Doppler ultrasound followed by DSA were performed to assess the radiological signs of cerebral vasospasm (CVS).

### Phenotype Alignment for Temporal Stratification of Samples

**Fig. 1:**
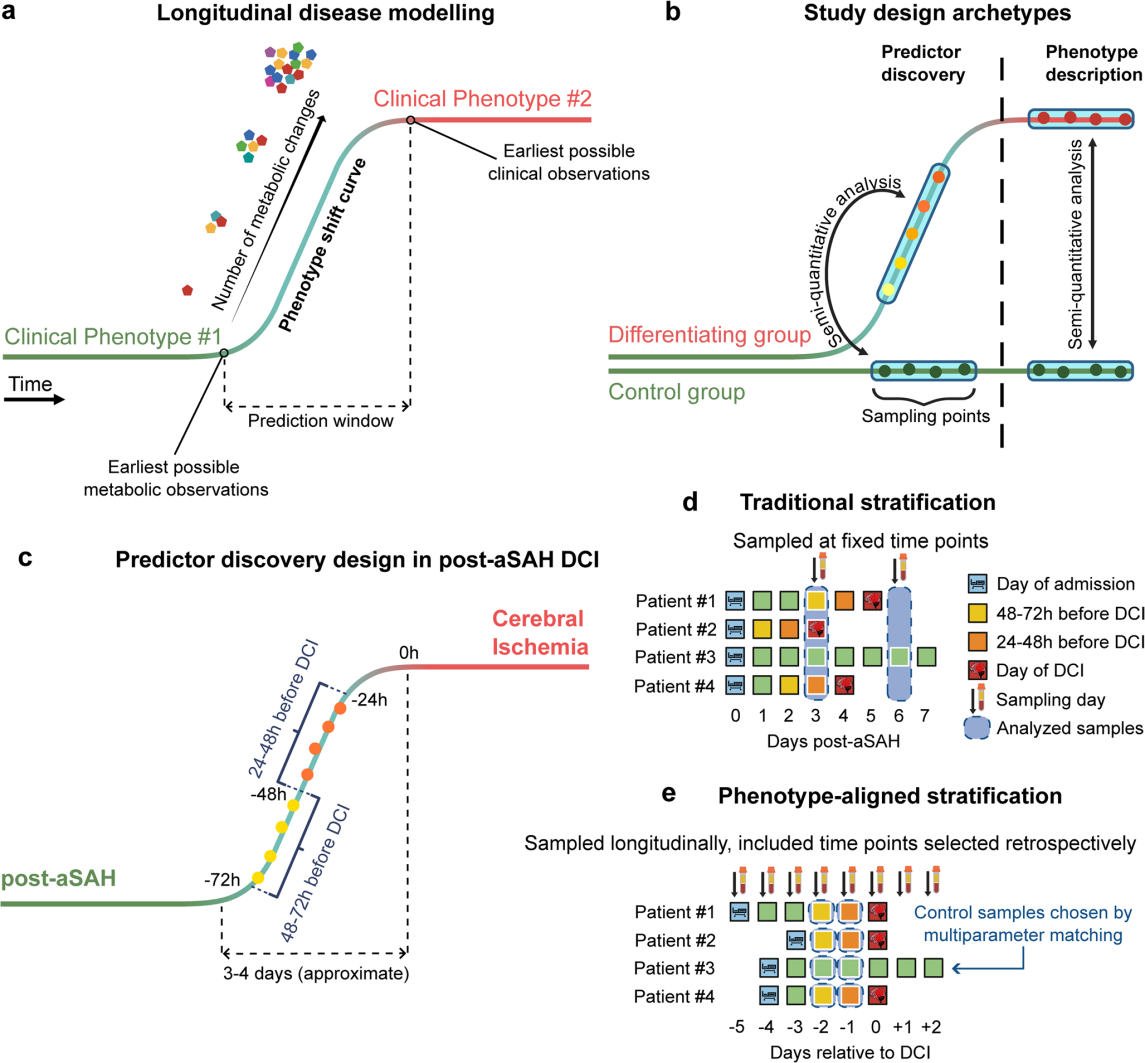
The Phenotype-Aligned Metabolomics Framework. Conceptual framework for phenotype-aligned metabolomics (PAM). (**a**) Three phases of phenotype transition: baseline, shift curve, and developed phenotype. (**b**) Discovery focus on metabolic predictors preceding phenotype shift versus descriptors of established phenotype. (**c**) Distribution of delayed cerebral ischemia (DCI) occurrence over time post-aSAH. (**d**) Fixed-timepoint sampling introduces temporal heterogeneity. (**e**) PAM aligns samples to individual event times

We developed the phenotype-aligned metabolomics (PAM) model to increase the likelihood of discovering actionable metabolic markers by targeting pre-event temporal windows. The framework comprises three conceptual phases: the baseline clinical phenotype, the phenotype shift curve representing the interval between earliest metabolic changes and first clinical manifestations, and the developed clinical phenotype (Fig. 1a). PAM operates on the principle that clinical features represent the culmination of preceding metabolic changes, enabling discovery of metabolic predictors leading to divergent clinical trajectories rather than descriptors of established phenotypes (Fig. 1b). The current implementation retrospectively aligns samples to individual clinical event times, correcting for inter-patient variability in onset timing while assuming similar disease progression across patients. In this study we estimated time-to-shift of post-aSAH DCI at 3–4 days (Fig. 1c). Samples were retrospectively aligned to individual DCI onset times rather than fixed calendar days.

Out of 41 patients who developed DCI in our cohort, 30 contributed available samples 24–72 h pre-DCI. These samples were stratified into two temporal windows based on time before DCI diagnosis: 24–48 h pre-DCI (*n* = 16 samples) and 48–72 h pre-DCI (*n* = 20 samples), for a total of 36 samples from 30 DCI patients. Clinical sampling schedules (predominantly every-other-day collection) resulted in most patients contributing to a single temporal window, with 6 patients sampled at both windows. This design provides independent temporal specificity across largely non-overlapping patient groups. Two control samples from control (no-DCI) patients were matched to each pre-DCI sample by day post-aSAH, age, sex, aneurysm location, Hunt-Hess, and mFisher scores, resulting in 53 samples from *n* = 30 control patients. The sample selection workflow is available as a chart in Supplementary Figure S1.

PAM addresses the analytical challenge of fixed-timepoint designs (Fig. 1d), where sampling at predetermined calendar intervals results in heterogeneous metabolic states. By aligning all DCI patients by the day of DCI onset, a cohesive baseline is formed for stratifying samples of interest which are equivalent in their temporal relationship to the clinical event (Fig. 1e).

### Sample Collection and Metabolomics

Fasting blood samples (5 ml) were collected in ethylenediaminetetraacetic acid (EDTA) tubes during the first 7 days post-aSAH. The samples were aliquoted, centrifuged at 7000 g for 10 min, and stored at -80 °C until extraction. Metabolomics were performed using a modified protocol reported previously [24]. Briefly, 50 µl plasma aliquots were thawed on ice and deproteinized with 200 µl LCMS-grade ice-cold methanol (MeOH) (VWR). After centrifugation at 5 °C and 18,000 g for 13-minutes, the supernatant was vacuum dried at 60 °C and stored at -80 °C for analysis. Before redispersion, dried samples were equilibrated at 5 °C for 15 min. Samples were redispersed in a 1:1 ratio of initial plasma volume to mobile phase A (0.1% formic acid), followed by centrifugation at 5 °C and 18,000 g for 13-minutes. 50 µl of ultrapure water underwent all extraction steps to serve as background cutoff. Quality control (QC) samples consisted of a mixed whole-sample pool. Extractions were performed in random order.

Samples were injected into a Vanquish ultra-high performance liquid chromatography (UHPLC) system coupled to an Exploris 480 Orbitrap mass spectrometer (MS) (Thermo Scientific) equipped with a heated electrospray ionization (H-ESI) source. A Hitachi L-6200 pump (Merck) was used to deliver 0.05% formic acid (ACS-grade, Sigma Aldrich) in LCMS-grade methanol post-column (VWR) at a gradient flowrate from 100 µl/min to 0 µl/min decreasing over 5.5 min. Metabolites were separated on a Kinetex F5 2.6 μm 100 × 2.1 mm 100 Å column (Phenomenex) at 250 µl/min flow rate, with elution conditions changing linearly between the following points: 0 min, 0% B; 0.6 min, 0% B; 3.9 min, 97% B; 5 min, 97% B; 5.01 min, 0% B; 1.99 min equilibration for a total run time of 7 min.

Source parameters were 3200 V and 2500 V voltage for positive and negative ionization respectively, 30 sheath gas, 8 aux gas, 325 °C ion transfer tube temperature, 230 °C vaporizer temperature. The method used scan parameters of 240,000 mass resolution (full width at half maximum at 200 m/z), 70–1000 m/z range, 60% RF lens, 300% (3e6) AGC target, 100 ms maximum injection time, using small molecule mode with internal calibration enabled in scan-to-scan mode. Fragmentation data was acquired via data-dependent shotgun MS2 at 60,000 precursor and 30,000 fragment mass resolutions using three ramped energies of 20%, 55%, and 80% (normalized collision energy). Injection volume was 5 µl.

### Data Processing

Data processing was performed in Compound Discoverer 3.3 (Thermo Fisher Scientific) and included retention time alignment, peak detection, feature reduction, quality control filtering, systematic error removal using random forest (SERRF) quality control [25], and metabolite annotation using ChemSpider, Human Metabolome Database (HMDB) [26], Kyoto Encyclopedia of Genes and Genomes (KEGG) [27] at 3 ppm mass tolerance, and fragment matching against the mzCloud spectral library (Thermo Scientific) at 10 ppm mass tolerance. Arginine, citrulline, and ornithine were confirmed at identification level 1 [28]. After duplicate removal, 9,135 total compounds (annotated and unannotated) were retained, out of which 2,022 were annotated.

### Statistical Analysis

All analyses used Python 3.9 + with statsmodels, scikit-learn, and scipy (two-sided tests, α = 0.05 unless otherwise specified). Metabolite peak areas were log₂-transformed for all analyses. Principal component analysis (PCA) was performed on the full dataset (annotated metabolites and m/z values for unknowns) after Pareto-scaling. Outliers were identified using Hotelling’s T² statistic (α = 0.001); one sample exhibited extreme deviation (T²=86.3, > 4× critical threshold) and was excluded, yielding 88 samples for analysis. For discovery analysis, independent two-sample t-tests with Benjamini-Hochberg FDR correction (q < 0.05) were applied to identify differential metabolites between temporal groups. Metabolite ratios (Arg/Orn, Arg/Cit, Gln/Glu) were calculated in log₂ space and tested using t-tests with FDR correction. To ensure independent observations in regression models, No-DCI samples were averaged within patients, yielding 45 independent observations (16 DCI, 29 No-DCI). Sensitivity analysis for this approach was performed using the Monte Carlo method (1000 iterations with random single-sample selection). Logistic regression models (arginine alone, mFisher grade alone, combined) were fit with standardized predictors (z-scores). Model performance was assessed using pseudo-R² (McFadden), AIC, and ROC analysis. AUC confidence intervals were estimated via bootstrap (2,000 iterations, stratified by outcome). Pairwise area under curve (AUC) comparisons used DeLong’s test. Correlations were assessed using Spearman’s rank correlation. All data visualization used matplotlib and seaborn packages. Statistical significance was defined as *p* < 0.05 for hypothesis tests and q < 0.05 for FDR-corrected analyses.

## Results

### Clinical Characteristics of Studied Patients

Out of the total 101 patients, *n* = 30 DCI patients contributed 36 samples within the 24–72 h pre-DCI window, to which 53 control samples from *n* = 30 no-DCI (control) patients were matched. The distribution of DCI occurrence for the 30 included DCI patients is shown in histogram form in Supplementary Fig. S2. For the 60 total included patients, the median age of the was 60 years (IQR: 47-64.25). Aneurysms were predominantly located in the anterior circulation (52 patients, 87%). On admission, the median Hunt and Hess grade was 3 (IQR: 2–3), and the median modified Fisher scale score was 4 (IQR: 3–4). DCI typically occurred at a median of 5 days post-hemorrhage (IQR: 4–5 days). CVS was detected in all DCI patients. Full characteristics are available in Table 1.

**Table 1:** Clinical characteristics of studied patients. DCI – Delayed cerebral Ischemia; IQR – interquartile range

Characteristic		Total (*n* = 60)	DCI (*n* = 30)	No DCI (*n* = 30)
Age [years]		60 (IQR: 47-64.25)	60.5 (IQR:47-64.75)	58.5 (IQR: 46.25-62)
Sex [female; n (%)]		30 (50%)	16 (53%)	14 (47%)
Aneurysm localization [anterior circulation; n (%)]		52 (87%)	27 (90%)	25 (83%)
Hunt and Hess scale		3 (IQR: 2–3)	3 (IQR: 3–3)	2 (IQR: 2–3)
Modified Fisher scale		4 (IQR: 3–4)	4 (IQR: 4–4)	3 (IQR: 2–4)
Intracerebral Hemorrhage [n (%)]		11 (18%)	7 (23%)	4 (13%)
Hydrocephalus [n (%)]		16 (26%)	12 (39%)	4 (13%)
DCI [n (%)]		30 (50%)	30 (100%)	Not applicable
Day of DCI		5 (IQR: 4–5)	5 (IQR: 4–5)	Not applicable
Glasgow Outcome Scale	At discharge	3 (IQR: 3–4)	3 (IQR: 3–3)	3 (IQR: 3–4)
	1 week	3 (IQR: 2–4)	3 (IQR:2–3)	4 (IQR: 3–4)
	1 month	4 (IQR: 2–4)	2 (IQR: 1.25-4)	4 (IQR: 3.25-4)
	12 months	4 (IQR: 2–4)	2 (IQR: 1.25-4)	4 (IQR: 3.25-4)

### Untargeted Analysis and Arginine Discovery

**Fig. 2:**
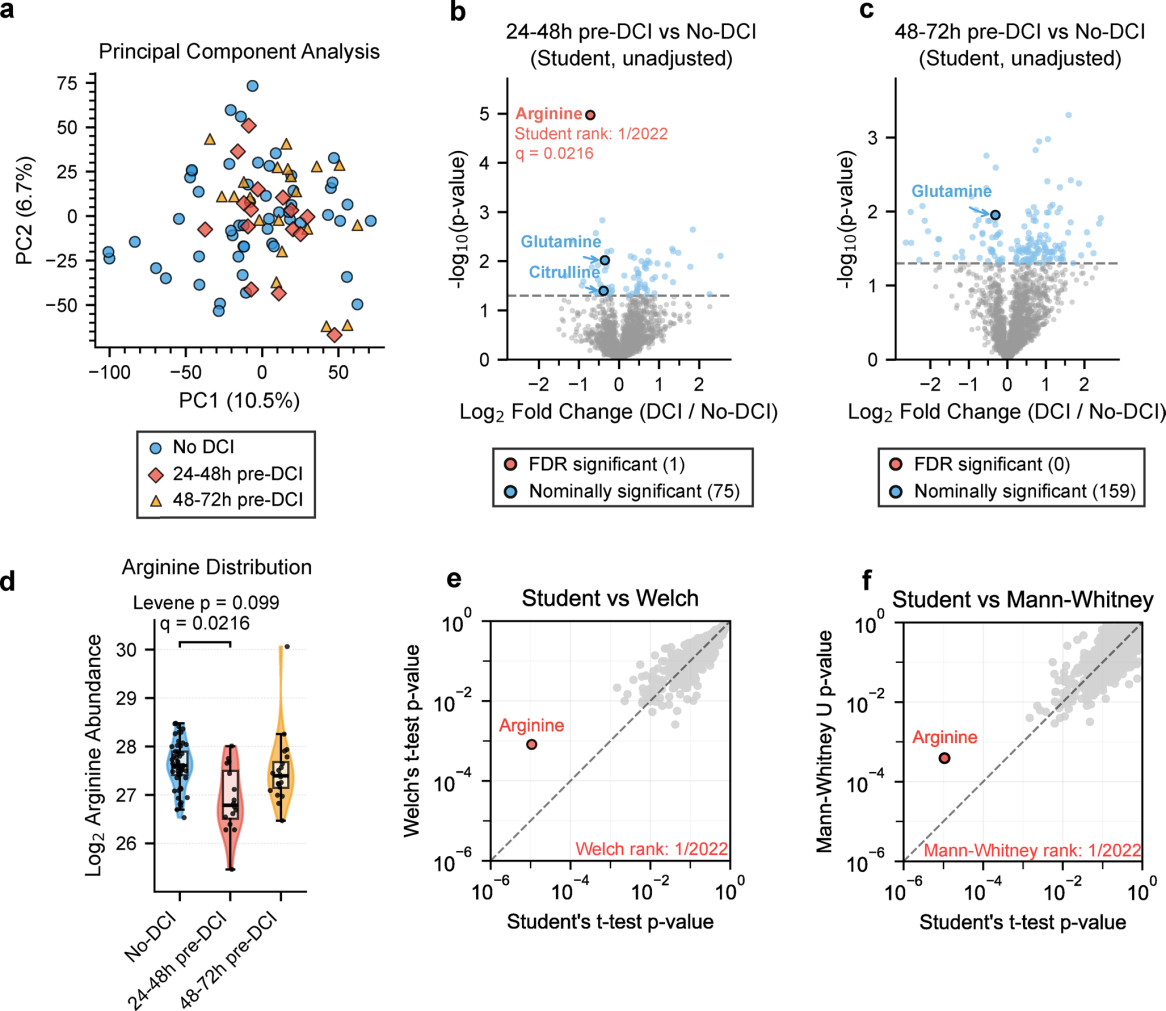
Arginine Discovery via Untargeted Metabolomic Analysis. Arginine is the top-ranked metabolite across three statistical methods. (**a**) Principal component analysis (PCA) of 88 plasma samples (9,135 features) shows no clustering by delayed cerebral ischemia (DCI) status without temporal stratification. (**b**, **c**) Volcano plots (Student’s t-test) at 24–48 h and 48–72 h pre-DCI; false discovery rate (FDR)-significant metabolites color-coded (arginine q = 0.022 at 24–48 h only). (**d**) Arginine distribution showing borderline variance heterogeneity (Levene’s *p* = 0.099). (**e**, **f**) Method comparison scatters: Student’s versus Welch’s and Mann-Whitney U tests; arginine ranks #1 of 2,022 metabolites

Principal component analysis (9,135 compounds, 88 samples) revealed no clustering by DCI status (Fig. 2a). The first five components accounted for 33.06% of total variance (PC1: 10.47%, PC2: 6.74%), with complete intermixing of DCI and No-DCI groups (full PCA matrix is available in Supplementary Fig. S3). The absence of global clustering indicates that DCI-predictive changes are metabolically specific rather than system-wide, supporting the rationale behind PAM design.

Comprehensive metabolomics screening (2,022 annotated metabolites) at 24–48 h pre-DCI identified 75 metabolites with nominal significance (*p* < 0.05, Student’s t-test) and one achieving FDR significance: arginine (q = 0.022, Fig. 2b). At 48–72 h pre-DCI, zero metabolites achieved FDR significance across any statistical method, and arginine showed no difference (*p* = 0.312, Fig. 2c), supporting temporal specificity of changes observed at 24–48 h.

Arginine exhibited 39% depletion in DCI patients at 24–48 h (log₂ fold-change = -0.715, mean log₂-transformed peak area 26.90 ± 0.68 in DCI vs. 27.61 ± 0.48 in No-DCI) with borderline variance heterogeneity (Levene *p* = 0.099, Fig. 2d). Borderline heteroscedasticity triggered the use of two additional statistical methods: Welch’s t-test (robust to unequal variance) *p* = 8.15 × 10⁻⁴ and Mann-Whitney U test (non-parametric, distribution-free) *p* = 3.89 × 10⁻⁴. Arginine ranked #1 of 2,022 metabolites across all three statistical tests (Fig. 2e, f).

Paired analysis for the 6 DCI patients with both timepoint measurements available followed a similar trend (Wilcoxon *p* = 0.063, log₂ fold-change = -0.70, Supplementary Fig. S4). Sensitivity analysis assessed the impact of two outliers visible in Fig. 2d: the lowest arginine value at 24–48 h pre-DCI and the highest at 48–72 h pre-DCI (Supplementary Fig. S5). For the 24–48 h window, significance was maintained after outlier removal (*p* = 6.82 × 10⁻⁵ vs. 1.07 × 10⁻⁵) with modestly attenuated effect size (35% vs. 39% depletion). Variance homogeneity improved (Levene’s *p* = 0.27 vs. 0.10), strengthening the validity of parametric assumptions. For the 48–72 h window, the signal became borderline (*p* = 0.054 vs. 0.460) with increased effect size (15% vs. 7% depletion).

In the full dataset (9,135 compounds regardless of annotation status), arginine remained FDR-significant with Student’s t-test (q = 0.0263) despite 4.5-fold increased multiple testing burden (Supplementary Fig. S6). Three additional unannotated features achieved significance at 24–48 h, two being arginine in-source fragmentation artifacts. One unknown metabolite was strongly upregulated (m/z 368.18908, q = 0.0263, log₂ fold-change = + 1.922). Zero features achieved FDR significance at 48–72 h, strengthening temporal specificity of the 24–48 h window.

Compared to a fixed-timepoint analysis of the cohort, PAM achieved at least 50 times stronger p-values for arginine along with the highest fold change, yielding the only FDR-significant analyte and demonstrating the advantage of stratification via PAM over conventional designs (Supplementary Table S1). The temporal distribution of arginine levels versus days post-aSAH is shown in Supplementary Fig. S7.

### Clinical Performance Assessment

**Fig. 3:**
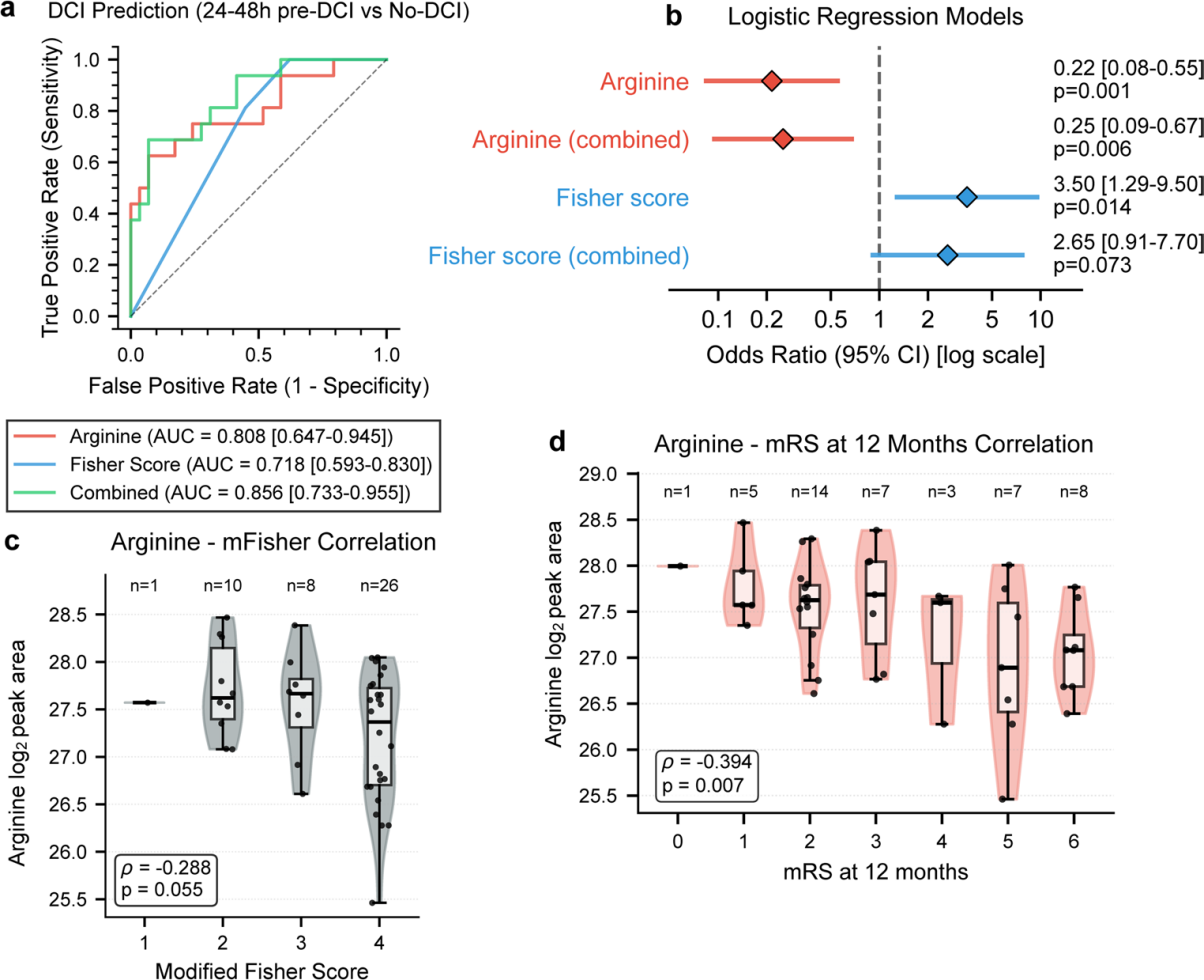
Assessment of Arginine as a DCI Predictor. Logistic regression validation of arginine predictive performance. (**a**) Receiver operating characteristic (ROC) curves with bootstrap 95% confidence intervals (2,000 iterations). Arginine alone: area under curve (AUC) 0.808; mFisher score: AUC 0.718; Combined model: AUC 0.856. mFisher versus Combined model was significantly different (DeLong’s test, *p* = 0.011). (**b**) Forest plot of logistic regression results. Arginine odds ratio (OR) 0.22 (95% CI: 0.08–0.55, *p* = 0.001). (**c**) Spearman Rank Correlation between arginine and modified Fisher score shows weak correlation (ρ=-0.29, *p* = 0.055), indicating that the two variables carry independent information. (**d**) Spearman Rank Correlation between arginine and modified Rankin Score at 12 months after discharge, showing statistically significant correlation at a moderate level (ρ=-0.394, *p* = 0.007). mRS – modified Rankin Scale

To assess clinical utility independent of the discovery framework, arginine was evaluated through logistic regression against the modified Fisher radiographic severity score. Using patient-averaged values to ensure independent observations, arginine outperformed the modified Fisher score in bootstrap-derived confidence intervals (2,000 iterations). Arginine achieved AUC 0.808 (95% CI: 0.647–0.945, Fig. 3a) compared to mFisher score AUC 0.718 (95% CI: 0.593–0.830). The combined model achieved AUC 0.856 (95% CI: 0.733–0.955). In univariate regression, arginine demonstrated odds ratio 0.22 (95% CI: 0.08–0.55, *p* = 0.001) and pseudo-R²=0.268, while mFisher showed odds ratio 3.50 (95% CI: 1.29–9.50, *p* = 0.014) and pseudo-R²=0.162 (Fig. 3b). In multivariable analysis, arginine maintained significance with little odds ratio change (odds ratio 0.25, *p* = 0.006) while mFisher lost statistical significance (odds ratio 2.65, *p* = 0.073). Full model diagnostics are available in Supplementary Fig. S8. Monte Carlo sensitivity analysis (1,000 iterations with random single-sample selection per control patient) confirmed robustness of the averaging approach for handling multiple measures in regression analysis (mean AUC 0.801 ± 0.13 versus 0.808 for averaged values; Supplementary Fig. S9).

**Fig. 4:**
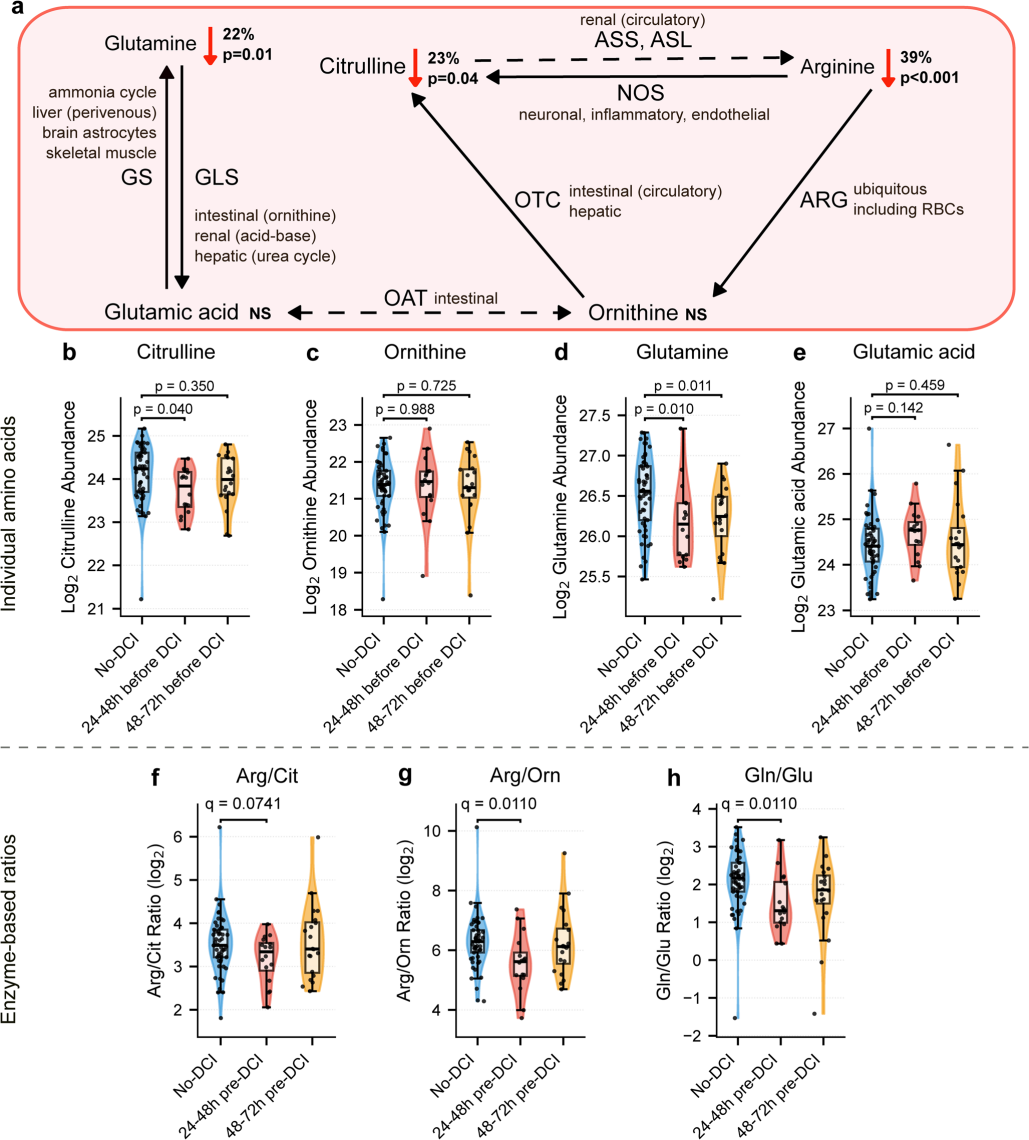
Arginine Pathway Dysregulation and Metabolite Ratios. Pathway-level investigation of arginine and glutamine metabolism. (**a**) Arginine-related metabolic pathways with compartmentalization. (**b**–**e**) Distribution of individual metabolites: citrulline (*p* = 0.040), ornithine (*p* = 0.988), glutamine (*p* = 0.010 at 24-48 h, *p* = 0.011 at 48-72 h), and glutamic acid (*p* = 0.142). (**f**–**h**) Metabolite ratios (log₂ space): arginine/citrulline (q = 0.074), arginine/ornithine (q = 0.011), and glutamine/glutamate (q = 0.011). Statistical significance for Arg/Orn and Gln/Glu achieved across three methods (Student’s t-test, Welch’s t-test, Mann-Whitney U test, Supplementary Fig. S12). RBCs – red blood cells, GS - Glutamine Synthetase, GLS - Glutaminase, OAT - Ornithine Aminotransferase, OTC - Ornithine Transcarbamylase, ARG - Arginase, ASS - Argininosuccinate Synthase, ASL - Argininosuccinate Lyase

DeLong’s test demonstrated that the combined model significantly outperformed mFisher alone (ΔAUC = 0.138, *p* = 0.011) but did not differ from arginine alone (ΔAUC = 0.047, *p* = 0.279). Weak correlation between the two variables (Spearman ρ=-0.29, *p* = 0.055) demonstrates arginine provides information independent of hemorrhage severity (Fig. 3c). Arginine also correlated with long-term functional outcome, showing moderate association with 12-month modified Rankin Scale scores (ρ=-0.394, *p* = 0.007; Fig. 4d). Consistent correlations emerged across Glasgow Outcome Scale assessments at discharge through 12 months (ρ = 0.364 to 0.514, all *p* < 0.02; Supplementary Fig. S10).

### Mechanistic Investigation

Pathway-level analysis examined whether arginine depletion occurred in isolation or reflected coordinate dysregulation of related amino acid metabolism. Following arginine discovery, hypothesis-driven mechanistic investigation examined arginine-related amino acids (Fig. 4a).

Citrulline was nominally depleted at 24–48 h (*p* = 0.040, log₂ fold-change = -0.38, Fig. 4b). Ornithine showed no change at either window (24–48 h: *p* = 0.988; 48–72 h: *p* = 0.725, Fig. 4c). Glutamine was depleted at both windows (24–48 h: *p* = 0.010, log₂ fold-change = -0.35; 48–72 h: *p* = 0.011, log₂ fold-change = -0.31, Fig. 4d). Glutamic acid showed no significant change (24–48 h: *p* = 0.142; 48–72 h: *p* = 0.459, Fig. 4e). None survived FDR correction as individual metabolites (Supplementary Fig. S11).

Three enzyme-based metabolite ratios were examined, all either reaching or approaching FDR significance (Supplementary Fig. S12). The arginine/citrulline ratio approached significance at 24–48 h (q = 0.074, Levene *p* = 0.797, Fig. 4f). At 48–72 h, the ratio showed no difference (*p* = 0.779). The arginine/ornithine ratio was significantly decreased at 24–48 h (q = 0.011, Levene *p* = 0.475, Fig. 4g). At 48–72 h, the ratio showed no difference (*p* = 0.892). Similarly, the glutamine/glutamate ratio was decreased at 24–48 h (q = 0.009, Levene *p* = 0.885, Fig. 4h). At 48–72 h this ratio showed a nominal trend (*p* = 0.053).

All three ratios exhibited minimal variance heterogeneity (Levene *p* > 0.4) and minimal p-value variation between methods (0.7–2.2× fold-change) compared to individual arginine (76× for Welch versus Student). Relevant p and q values are available in heatmap format for individual amino acids (Supplementary Fig. S11) and ratios (Supplementary Fig. S12).

## Discussion

This study identifies plasma arginine as a candidate for metabolic prediction of DCI with pre-event temporal specificity. Comprehensive untargeted metabolomics identified arginine as the top-ranked predictor (ranked #1 of 2,022 metabolites) across all statistical methods. Independent validation through logistic regression confirmed predictive performance, outperforming the modified Fisher radiographic severity score. Mechanistic investigation revealed FDR-significant arginine/ornithine and glutamine/glutamate ratios consistent with arginase activity and coordinate metabolic dysregulation. Temporal specificity replicated across largely independent patient groups at each timepoint defines a narrow pre-event detection window of 24–48 h pre-DCI.

Previous studies have identified arginine dysregulation in DCI and CVS, albeit with critical temporal and sampling limitations. CSF-based studies demonstrated that decreased arginine/ornithine ratios measured in early post-SAH samples (days 1–3) predicted subsequent vasospasm development [15, 16], establishing the relevance of the arginine pathway. Untargeted plasma metabolomics studies, however, failed to identify arginine as an individual predictor [19, 21]. This study identified circulatory arginine as a single-molecule candidate predictor via temporal alignment. Direct comparison confirmed this approach was essential: fixed-timepoint analysis yielded p-values at least 50-fold weaker, failing to achieve FDR significance (Supplementary Table S1). This plasma-based finding also offers practical advantages over CSF monitoring: minimally invasive sampling, feasibility for serial measurements, and compatibility with standard clinical workflows.

Arginine depletion with concurrent nominal citrulline reduction (*p* = 0.040) and unchanged ornithine levels indicates impaired nitric oxide (NO) synthesis. The decreased arginine/ornithine ratio indicates increased arginase activity competing with endothelial nitric oxide synthase (eNOS) for arginine as a substrate. Arginase-1 released from lysed erythrocytes following hemorrhage has been suspected to deplete arginine in CSF [15], and our plasma findings corroborate this mechanism in circulation.

Increased arginase activity impairs endothelial function through multiple mechanisms. Arginine depletion directly limits NO synthesis, and substrate depletion triggers eNOS uncoupling where the enzyme produces reactive oxygen species rather than NO. Reduced NO availability is likely a primary mechanism impairing endothelial function and promoting vasoconstriction, creating conditions conducive to DCI development [29, 30].

Similar arginase-driven mechanisms occur in hemolytic disorders (sickle cell anemia, thalassemia), where arginase released from lysed erythrocytes causes vasoconstriction and pulmonary hypertension through arginine depletion [31, 32]. This parallel suggests potential conserved pathophysiological pathways linking hemolysis, arginase activity, and vascular dysfunction, though direct mechanistic verification in aSAH is needed.

Concurrent nominal citrulline depletion (*p* = 0.040, not FDR-significant) may reflect impaired NO pathway function, as citrulline is both a product of NO synthase activity and a substrate for arginine resynthesis via the arginine-citrulline cycle. However, unaltered ornithine levels (*p* = 0.988) suggest that ornithine is being rapidly metabolized or that arginase-mediated arginine-to-ornithine conversion is balanced by ornithine utilization.

These findings corroborate CSF observations of reduced arginine/ornithine ratios in DCI patients [15, 16], though prior studies employed wide sampling intervals without temporal alignment to pre-event windows, limiting their pre-event specificity.

Parallel dysregulation of glutamine/glutamate metabolism suggests coordinate metabolic stress beyond arginine pathway dysfunction alone. Glutamine depletion at both pre-DCI windows may reflect sustained metabolic demand in patients progressing toward DCI. Glutamine depletion is well-characterized in critical illness, driven by increased metabolic demand exceeding endogenous production capacity. During acute stress, inflammatory responses accelerate protein catabolism, depleting glutamine pools despite its status as the most abundant free amino acid in plasma [33, 34]. The observation of glutamine depletion in pre-DCI patients is consistent with general critical illness mechanisms, though whether this reflects DCI-specific pathophysiology or broader systemic stress responses remains unclear. Alterations in arginine, citrulline, and glutamine have been observed across various critical illnesses (sepsis, major surgery, acute lung injury) [35–37], suggesting systemic rather than organ-specific metabolic dysregulation.

The relationship between glutamine and arginine metabolism involves complex biosynthetic pathways that remain incompletely characterized. Glutamine may contribute to citrulline biosynthesis, and citrulline is in turn required for renal arginine production [38]. While methodological constraints have limited definitive characterization of this glutamine-arginine axis [38], our observation of parallel depletion is consistent with potential biosynthetic linkage. Thus, impaired biosynthesis or absorption of these amino acids currently cannot currently be ruled out as contributing factors. Kidney function showed no indication of impairment in our DCI patients on admission, though this assessment lacked longitudinal follow-up and severe renal dysfunction was an exclusion criterion in our study (Supplementary Data S1).

Plasma arginine occupies a distinct position in the DCI biomarker landscape. CSF-based biomarkers, including arginase-1, arginine/ornithine ratios, and dimethylarginines (ADMA, SDMA), have established the biological relevance of arginine pathway dysregulation in DCI [15–18] but require invasive sampling via external ventricular drain or lumbar puncture, limiting serial monitoring and broader implementation. Although prophylactic lumbar drainage following the EARLYDRAIN [39] trial may improve CSF accessibility, plasma sampling remains more universally feasible for serial monitoring. Matricellular proteins (periostin, osteopontin, galectin-3) identified through machine learning models show promise but remain investigational and require validation [40]. Transcranial Doppler and EEG-based biomarkers provide real-time physiological monitoring but require continuous measurement and specialized expertise [41, 42].

Plasma arginine offers key advantages: minimally invasive sampling compatible with serial monitoring, pre-event detection in the 24–48 h window enabling proactive rather than reactive intervention, and single-molecule simplicity suitable for clinical assay development. These features position arginine as a translatable biomarker candidate bridging the gap between CSF biology and practical clinical implementation. While Chikh et al. identified arginine pathway dysregulation through enrichment analysis of a 20-metabolite predictive panel requiring comprehensive LC-MS profiling [19], plasma arginine as a single-molecule biomarker offers a translatable alternative readily implementable through established clinical assay platforms.

Clinical translation of plasma arginine as a DCI predictor requires prospective validation. Arginine measurement is feasible using liquid chromatography-tandem mass spectrometry (LC-MS) platforms already deployed in many clinical laboratories, or enzymatic assays currently employed for metabolic disease screening (arginase deficiency, urea cycle disorders), with turnaround times suitable for clinical decision-making. A practical implementation strategy would involve daily plasma sampling during the DCI risk window (Days 3–14 post-aSAH) integrated into routine neurocritical care phlebotomy schedules. Declining arginine levels or low arginine/ornithine ratios could trigger clinical decision points: optimization of blood pressure targets to maximize cerebral perfusion, initiation of prophylactic vasodilator therapy, or escalation to intensified monitoring protocols. Current DCI surveillance relies on clinical examination and transcranial Doppler, which are reactive measures identifying vasospasm after onset. A 24–48 h pre-event biomarker enables proactive intervention during the metabolic prodrome, potentially preventing progression to clinical DCI.

Arginine supplementation represents a mechanistically rational but unproven therapeutic approach. Prior trials of arginine supplementation in critical illness have shown mixed results, with some studies reporting harm in patients with severe sepsis and shock [43, 44]. The distinct aSAH pathophysiology (hemolysis-driven arginase release causing localized arginine depletion in the subarachnoid space) and defined 24–48 h therapeutic window warrant investigation. Earlier initiation of established preventive measures guided by arginine levels represents the most immediate clinical application. Arginase inhibitors remain experimental but represent another mechanistically rational therapeutic target [45].

The primary limitation of current findings is sample size. While appropriate for discovery-phase metabolomics, multi-center validation in larger cohorts is essential before clinical implementation. The single-center design limits assessment of generalizability across institutions with different patient populations and clinical practices. Wide bootstrap confidence intervals reflect uncertainty from the discovery sample size. DCI cohorts are characteristically small due to the condition’s moderate incidence and the requirement for intensive longitudinal sampling. Our cohort size is consistent with prior metabolomics studies in this population. A two-center validation cohort is currently underway. Prospective validation will assess predictive performance, optimal cutoff thresholds, and clinical utility in real-time prediction. Decision curve analysis and prospective impact studies will be necessary to demonstrate clinical utility beyond predictive performance metrics, including assessment of intervention effectiveness when guided by arginine levels.

A secondary limitation is the seven-day sampling window, which excludes late-onset DCI occurring beyond this timeframe. This temporal constraint likely accounts for the earlier-than-typical DCI onset distribution observed in our cohort (median day 5, IQR 4–5, Supplementary Fig. S2).

The absence of global metabolic separation in principal component analysis underscores the value of phenotype-aligned temporal stratification: arginine-specific metabolic divergence emerged through stratification by temporal relationship to individual DCI onset. This temporal stratification approach is applicable to any condition with discrete clinical events and can be applied to existing longitudinal omics datasets at no additional experimental cost.

The current study employed retrospective stratification targeting predefined temporal windows rather than comprehensive longitudinal characterization. The observed temporal specificity (significant at 24–48 h, non-significant at 48–72 h) supports genuine temporal dynamics rather than statistical artifact, however ideal validation would sample all available timepoints to characterize complete trajectories without a priori assumptions.

Additionally, twice-daily sampling should be considered in future studies to reduce temporal heterogeneity within pre-event windows, as 24-hour intervals may incompletely capture rapid metabolic shifts characteristic of critical illness. In the current study we avoided assessing the 0–24 h pre-DCI time window due to high expected volatility. With twice-daily sampling, assessing 0–12 h and 12–24 h windows would become possible, ameliorating this limitation.

Moreover, mechanistic interpretations are inferred from observational data rather than directly tested. Direct measurement of arginase activity, NOS function, and isotopic tracing of amino acid metabolism would provide mechanistic confirmation. Interventional trials are needed to establish causality and therapeutic potential.

## Conclusion

This study identifies plasma arginine as a metabolic predictor of DCI with a 24–48 h pre-event detection window, validated across multiple statistical methods and independent logistic regression. The finding corroborates CSF studies demonstrating arginine pathway dysregulation in DCI, while advancing translational feasibility through minimally invasive plasma sampling compatible with serial monitoring and standard clinical workflows. Clinically, arginine outperformed the modified Fisher radiographic severity score, demonstrating that metabolic profiling adds independent predictive information and enables individual-level pre-event detection rather than population risk stratification. Methodologically, phenotype-aligned temporal stratification reduced biological heterogeneity to reveal metabolic signals specific to pre-event metabolic phenotypes, which previously remained confounded by system-level changes. The PAM approach may aid in biomarker discovery for diseases where timely prediction of clinical onsets is critical.

## Supplementary Information

Below is the link to the electronic supplementary material.

Supplementary Material 1

## Data Availability

Raw metabolomics data is available in the figshare repository under the following DOI: 10.6084/m9.figshare.30813941. Filtered data is available in .csv format for 2,022 annotated metabolites and 9,035 unannotated+annotated metabolites in: 10.6084/m9.figshare.30813929.

## Abbreviations

ACN: acetonitrile
ADMA: Asymmetric Dimethylarginine
AGC: automatic gain control
Arg: arginine
aSAH: aneurysmal subarachnoid hemorrhage
AUC: area under curve
CI: confidence interval
Cit: citrulline
CSF: cerebrospinal fluid
CT: computed tomography
CV: coefficient of variation
CVS: cerebral vasospasm
DCI: delayed cerebral ischemia
DSA: digital subtraction angiography
EDTA: ethylenediaminetetraacetic acid
EEG: electroencephalogram
eNOS: endothelial nitric oxide synthase
FDR: false discovery rate
Gln: glutamine
Glu: glutamic acid
H: ESI–heated–electrospray ionization
HMDB: Human Metabolome Database
IQR: interquartile range
KEGG: Kyoto Encyclopedia of Genes and Genomes
LCMS: liquid chromatography–mass spectrometry
MeOH: methanol
MS: mass spectrometry
NO: nitric oxide
NOS: nitric oxide synthase
Orn: ornithine
PAM: phenotype–alignment model
PCA: principal component analysis
QC: quality control
RBCs: red blood cells
ROC: receiver operating characteristic
SD: standard deviation
SDMA: Symmetric Dimethylarginine
SERRF: Systematic Error Removal using Random Forest
UHPLC: ultra–high performance liquid chromatography
