# Supplementary material for: Phenotype-Aligned Metabolomics Identifies Plasma Arginine as a Candidate Predictor of Delayed Cerebral Ischemia after Aneurysmal Subarachnoid Hemorrhage
Source: Transl Stroke Res. 2026 Mar 25;17(2):35. doi: 10.1007/s12975-026-01421-0 (PMC13013107; doi:10.1007/s12975-026-01421-0)
Supplement: Supplementary file 1 — Supplementary Material 1 [file 12975_2026_1421_MOESM1_ESM.pdf]

Phenotype-Aligned Metabolomics Identifies Plasma Arginine as a Candidate Predictor of Delayed Cerebral Ischemia after Aneurysmal Subarachnoid Hemorrhage

Krzysztof Urbanowicz, Karol Wiśniewski, Mikołaj Opiełka, Michał Bieńkowski, Marta Popęda, Oliwier Krajewski, Ernest J. Bobeff, Karol Zaczkowski, Bartosz Szmyd, Dariusz J. Jaskólski, Ryszard T. Smoleński

Supplementary Information

The following inclusion and exclusion criteria were established:

<u>Inclusion criteria:</u>

1. Age between 18–75 years;
2. Single ruptured saccular intracranial aneurysm;
3. Treatment at the Department of Neurosurgery with microsurgery/endovascular technique;
4. Diagnosis and treatment within 1 day after aneurysm rupture;
5. Hunt–Hess grade between I and IV.

<u>Exclusion criteria:</u>

1. Age <18 or >75 years;
2. Multiple or familial aneurysms;
3. No surgical or endovascular treatment;
4. Diagnosis/treatment >1 day after aneurysm rupture;
5. Hunt–Hess grade V;
6. Chronic organ failure: heart (NYHA III/IV), kidney (GFR < 30 mL/1.73 m^2/min), liver (Child–Pugh score C/D);
7. Medical history of diabetes mellitus, neurodegenerative disease, or cardiovascular disease;
8. Pregnancy;
9. Coagulation disorders;
10. Legal incapacitation.

Supplementary Data S1: Inclusion and exclusion criteria used for patient recruitment

Supplementary Table S1: Comparison between fixed timepoint analysis and PAM stratification. PAM achieved 50-time stronger p-value than the statistically strongest Day 3 fixed timepoint variant while improving on effect size. PAM also yielded the only FDR-significant q-value after Benjamini-Hochberg correction in untargeted analysis. Log2FC - log2 fold change, BH – Benjamini-Hochberg, PAM – Phenotype Aligned Metabolomics, DCI – delayed cerebral ischemia

Analysis	Total n	DCI n	N DCI n	Arginine Log2FC	Arginine p Student	Arginine q BH
Days 1-3	63	26	37	-0.469	0.0007	0.248
Days 4-6	26	10	16	-0.138	0.6651	0.953
Day 3 only	46	19	27	-0.604	0.0005	0.538
All pooled	89	36	53	-0.378	0.0049	0.575
PAM 24-48h*	45	16	29	-0.715	1.07E-05	0.022

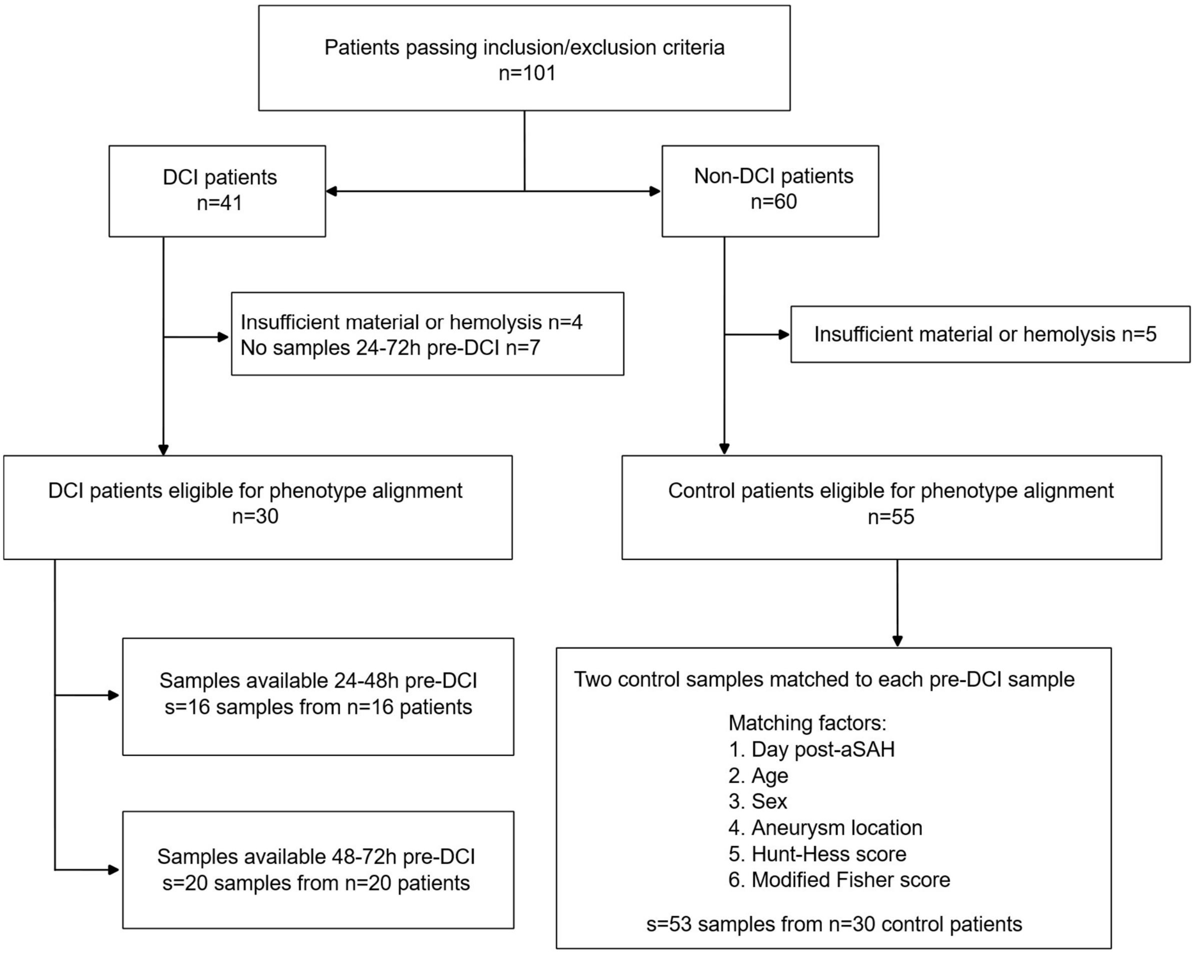

Supplementary Fig. S1: CONSORT-style chart for plasma sample selection in this study. For each pre-DCI sample, two control (no-DCI) samples were matched based on the listed criteria. Some overlap was possible between timepoints, resulting in 53 control samples selected for comparative analysis

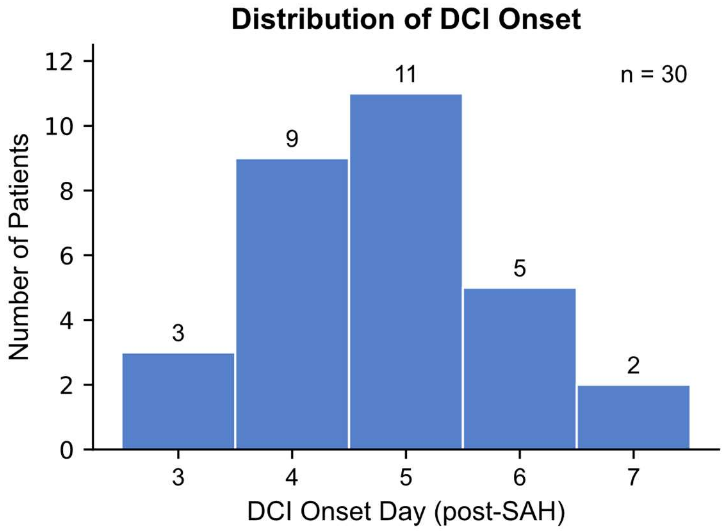

Supplementary Fig. S2: Histogram of DCI onset distribution for the 30 DCI patients included in discovery analysis. Peak of DCI in this study was day 5 (IQR 4-5) due to the 7-day sampling cutoff

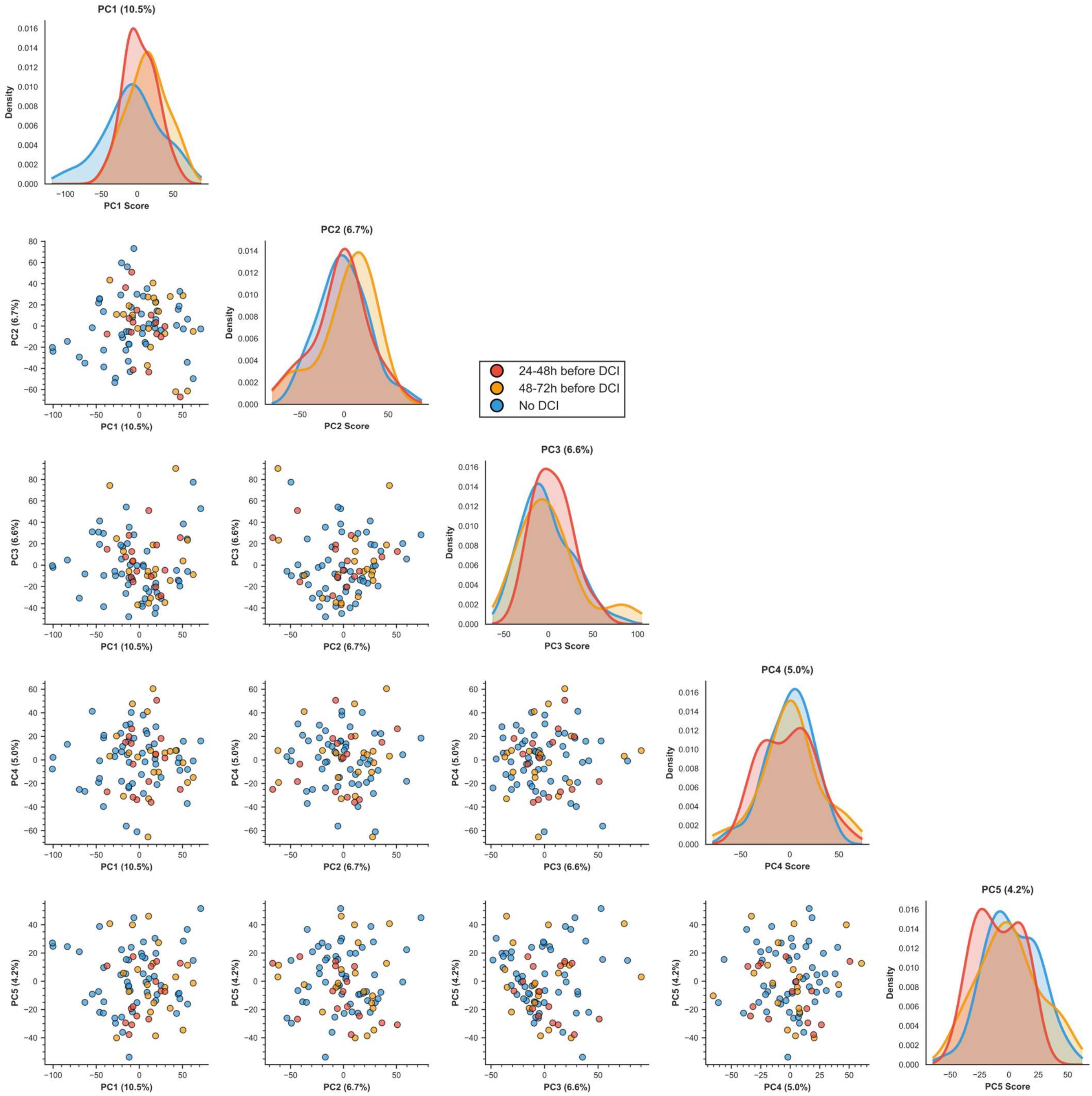

Supplementary Fig. S3: Principal Component Analysis – five-component matrix. Five-component principal component analysis (PCA) matrix with kernel density plots. Pareto-scaled data from 88 plasma samples (9,135 metabolite features). Groups: 24-48h pre-DCI (delayed cerebral ischemia, n=15), 48-72h pre-DCI (n=20), No-DCI (n=53). Complete intermixing across all principal component pairs demonstrates absence of global metabolic separation

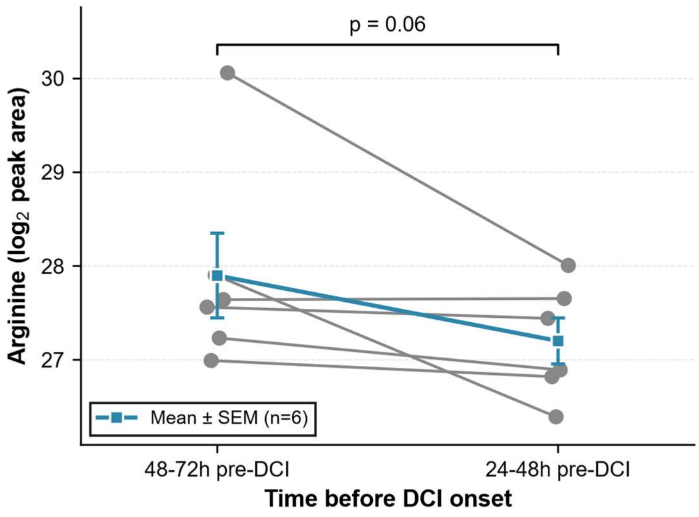

Supplementary Fig. S4: Paired analysis for 6 patients with both pre-DCI timepoint samples available. The paired analysis was trending at p=0.0625 (Wilcoxon Signed Rank test) and $\log_2$ fold-change = -0.70, albeit definitive conclusions would require more paired measurements to increase statistical power

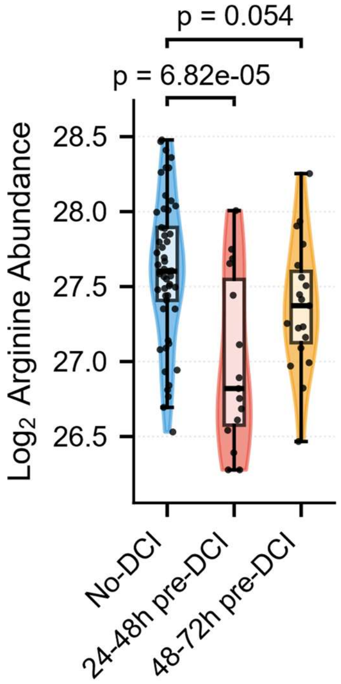

Supplementary Fig. S5: Sensitivity analysis for two outliers observed in the arginine distribution. Removing the lowest outlier in the 24-48h timepoint resulted in an increase to its p-value from p = 1.07×10^{-5} to 6.82×10^{-5}, maintaining significance with a modest reduction to effect size (39% to 35% depletion), while Levene p-value increased from 0.099 to 0.268, improving homoscedascity. For the 48-72h timepoint, the removal of its highest outlier improved the p-value from 0.460 to 0.054, however the effect size remained small (7% depletion to 15% depletion)

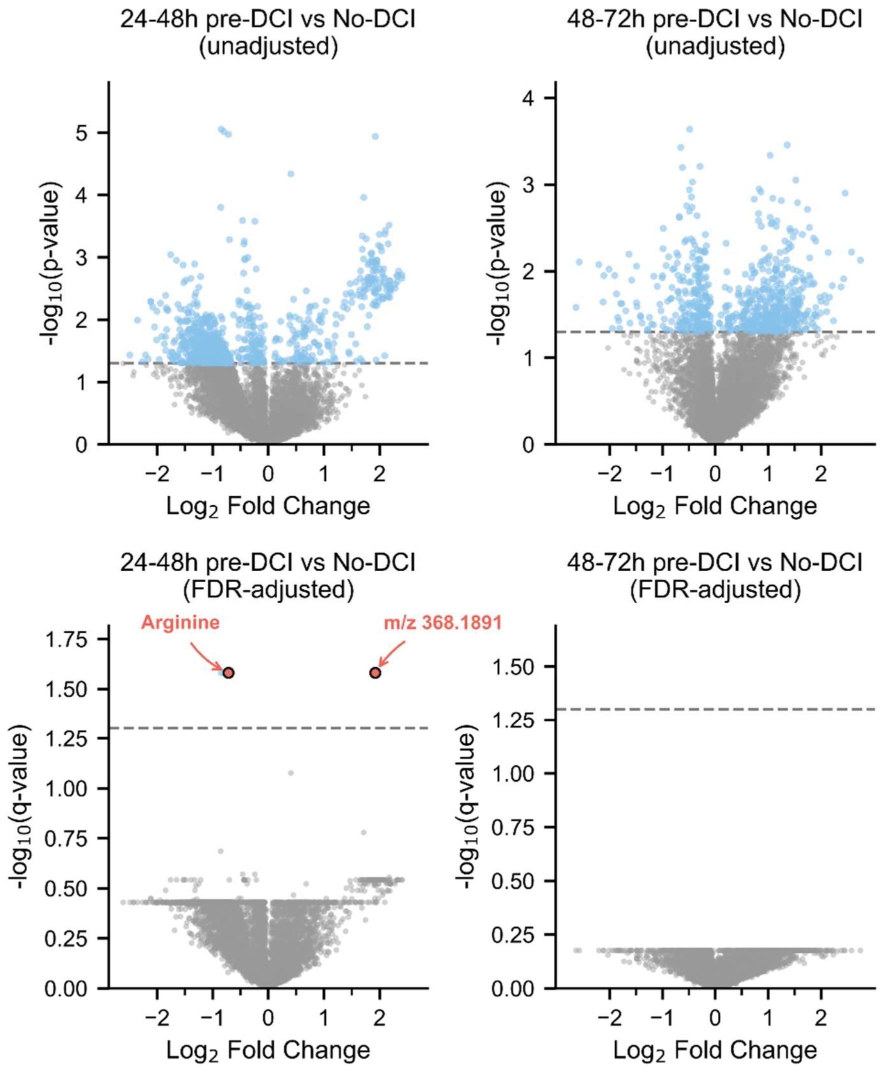

Supplementary Fig. S6: Volcano plots of the full dataset with unannotated compounds included. Comprehensive metabolite analysis including 9,135 compounds (annotated and unannotated) using Student's t-testing. (a,b) Unadjusted p-values at 24-48h and 48-72h pre-DCI (delayed cerebral ischemia). (c,d) False discovery rate (FDR)-adjusted q-values. Arginine and one unknown metabolite (m/z 368.1891) highlighted. Arginine survives 4.5× increased multiple testing burden. Zero features achieved FDR significance at 48-72h

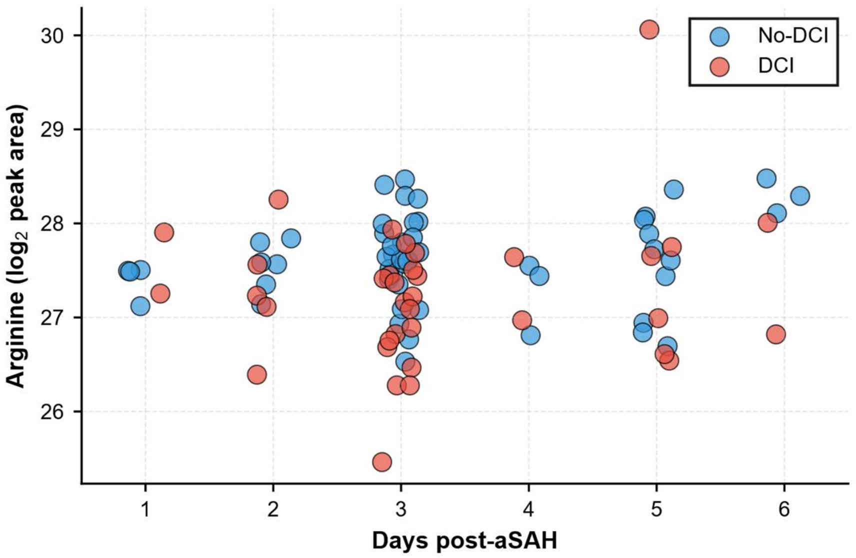

Supplementary Fig. S7: Post-aSAH day (fixed timepoint) distribution of arginine levels within the cohort. The high density visible on day 3 is the result of predominantly every-other-day sampling regime

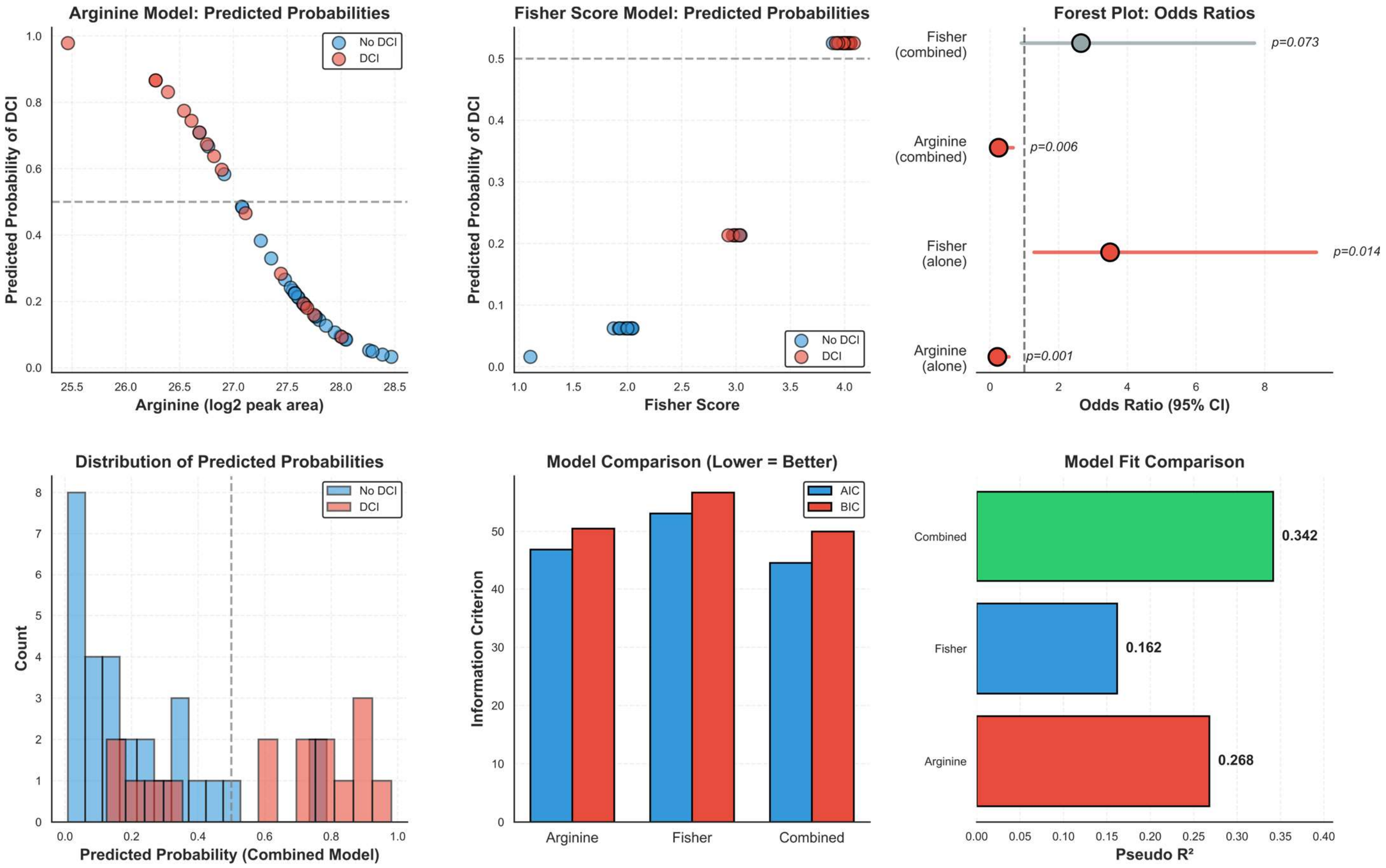

Supplementary Fig. S8: Logistic regression model diagnostics. Model diagnostics for arginine, mFisher score, and combined logistic regression models. Residual plots, influence diagnostics (Cook's distance, leverage), and model fit assessment. Standardized predictors used (z-scores). Dataset: 45 independent observations (16 DCI 24-48h before event, 29 No-DCI) after patient-level averaging to ensure independent observations

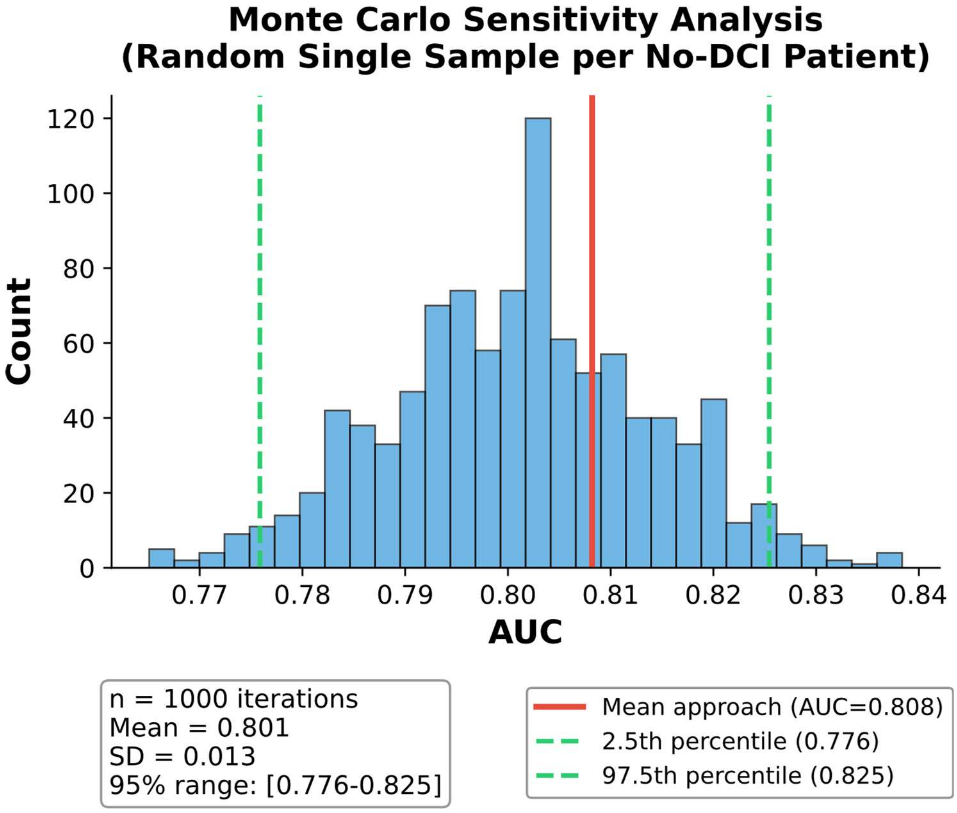

Supplementary Fig. S9: AUC distribution histogram of 1000 iterations for single-sample selection among control patients. Monte Carlo sensitivity analysis was performed to assess the robustness of the chosen averaging approach for handling of repeated measures in control patients. After 1000 iterations selecting random control patients, the mean AUC was 0.801 with 0.013 standard deviation, while the chosen averaging approach resulted in AUC 0.808, within range of the Monte Carlo Mean±SD. AUC – area under the curve, SD – standard deviation

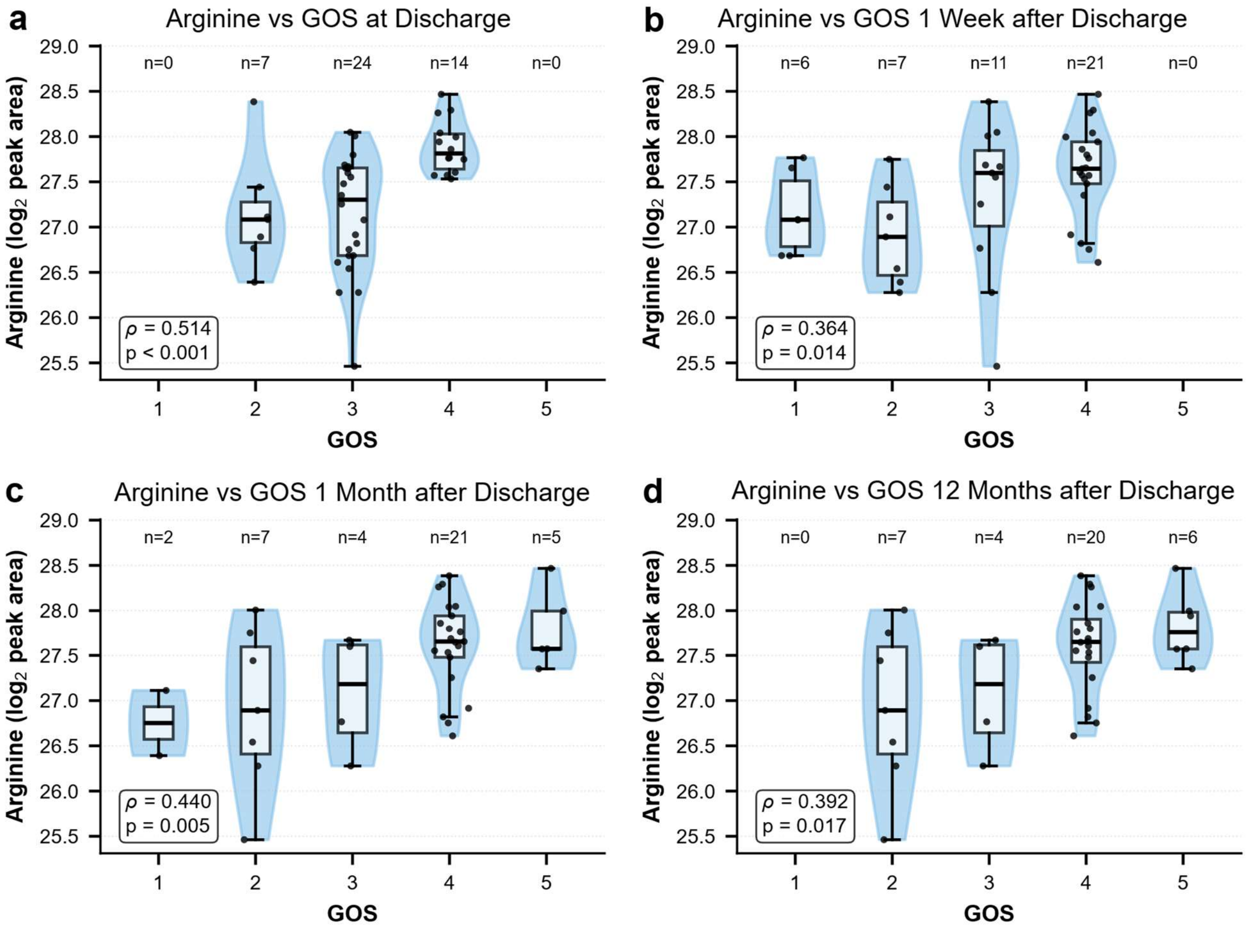

Supplementary Fig. S10: Spearman Correlations between 24-48h pre-DCI arginine levels and Glasgow Outcome Scale at four timepoints. Weak-to-moderate correlations between arginine 24-48h before DCI and GOS were observed at (a) discharge, (b) 1 week after discharge, (c) 1 month after discharge, and (d) 12 months after discharge. All p-values achieved significance. GOS – Glasgow Outcome Scale

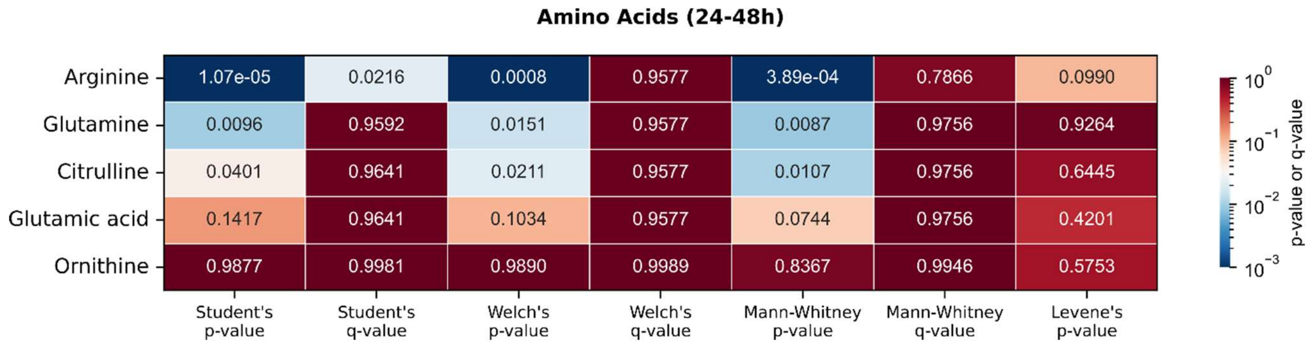

Supplementary Fig. S11: Amino acids statistical methods comparison. Heatmap comparing five amino acids across three statistical methods. Rows: arginine, citrulline, ornithine, glutamine, glutamic acid. Columns: Student's t-test, Welch's t-test, Mann-Whitney U test. Displays actual p-values (not log-transformed) with Levene's p-values for variance testing. Arginine is only metabolite achieving false discovery rate (FDR) significance (q=0.0216 with Student's t-test). DCI: delayed cerebral ischemia

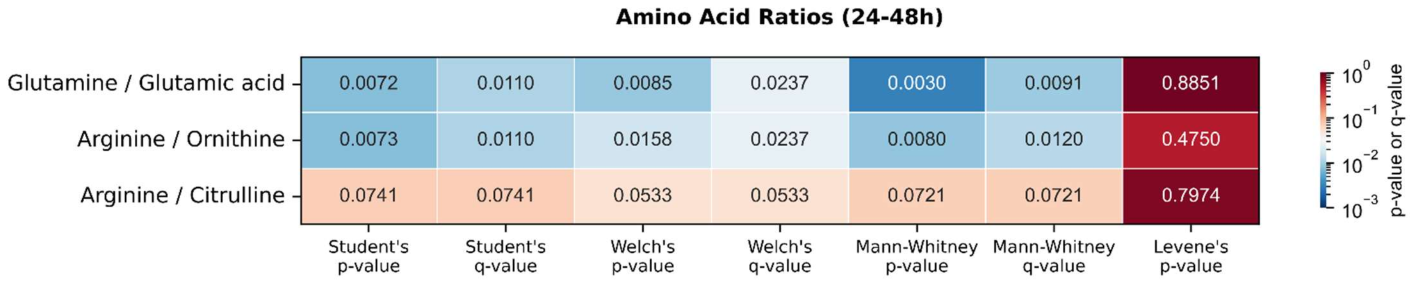

Supplementary Fig. S12: Metabolite ratios statistical methods comparison. Heatmap comparing three metabolite ratios across three statistical methods. Rows: arginine/ornithine, glutamine/glutamate, arginine/citrulline. Columns: Student's t-test, Welch's t-test, Mann-Whitney U test. Displays actual p-values with Levene's p-values (all >0.4). Arginine/ornithine and glutamine/glutamate achieve false discovery rate (FDR) significance (q<0.05) across all methods. Demonstrates excellent variance homogeneity and consistent ranking. DCI: delayed cerebral ischemia
